# Effects of a Multimodal Preparation Package on Vital Signs of Patients Waiting for Coronary Angiography

**DOI:** 10.17795/nmsjournal17518

**Published:** 2014-04-17

**Authors:** Mohsen Adib Hajbaghery, Tayebeh Moradi, Raheleh Mohseni

**Affiliations:** 1 Trauma Nursing Research Center, Faculty of Nursing and Midwifery, Kashan University of Medical Sciences, Kashan, IR Iran; 2 Department of Medical Surgical Nursing, Kashan University of Medical Sciences, Kashan, IR Iran

**Keywords:** Coronary Angiography, Patient Education, Vital Signs

## Abstract

**Background:**

Patients waiting for coronary angiography are often anxious and worried, experiencing considerable emotional problems before the procedure, which can result in an increase in blood pressure (BP), heart rate, respiratory rate and the myocardial oxygen demand. Such maladaptive responses may not only increase the patients need for sedative drugs, but also could increase the length of post angiography hospitalization. Therefore, it is important to implement some supportive actions to decrease the patients’ anxiety and to stabilize their vital signs before coronary angiography.

**Objectives:**

This study aimed to investigate the effects of a multimodal preparation package on vital signs of patients undergoing coronary angiography.

**Patients and Methods:**

A matched trial was conducted on 66 patients waiting for coronary angiography. Patients were assigned in intervention (n = 33) and control (n = 33) groups. A multimodal preparation package was implemented in intervention group, two hours before angiography. The data collection instrument consisted of questions on demographic characteristics and a table for recording the patients’ vital signs including systolic BP (SBP) and diastolic BP (DBP), heart rate, respiratory rate and body temperature. Vital signs were measured three times, the day before angiography, 30 minutes before and 30 minutes after the angiography, using a thermometer and a monitoring device. Data analysis was performed using the Kolmogo-Smirnov test, t test and Mann-Whitney U test.

**Results:**

From the total number of 66 patients, the 63.3% were male and married. No significant differences were observed between the mean of SBP and DBP and also the heart rate in the intervention and control groups, on the day before angiography. However, the mean SBP and DBP and heart rate of the intervention group were significantly lower compared to the control group, both 30 minutes before and 30 minutes after angiography. The intervention did not significantly change the respiration rate and temperature in the intervention group.

**Conclusions:**

The study showed that preparation package was effective in decreasing SBP and DBP, as well as heart rate. Therefore, using multimodal comprehensive preparation packages, such as the package used in the present report, is suggested.

## 1. Background

In recent years, invasive diagnostic procedures, such as coronary angiography, are increasingly used, as a result of an increase in cardiovascular disease incidence (1). Annually, more than one million cardiac catheterizations and coronary angiographies are performed in the United States alone (2). Patients waiting for coronary angiography are often anxious and worried (3), and experience a considerable emotional stress before the procedure (4). An investigation has shown that over 82% of patients waiting for coronary angiography are anxious (5). Anxiety can affect the patient's physiologic and biochemical responses. Anxiety activates the sympathetic nervous system and increases epinephrine and norepinephrine release. Blood pressure (BP), heart rate, respiratory rate and the myocardial oxygen demand would consequently increase. Also, it has been shown that the cardiac workload and the risk for ischemia and dysrhythmia are increased during coronary angiography (3, 6-9). Such maladaptive responses may not only increase the patients demand for sedative drugs, but would also increase the length of post angiography hospitalization (3, 10). Therefore, it is important to implement some supportive actions to decrease the patients’ anxiety and to stabilize their vital signs before coronary angiography (11).

Different pharmacologic and non-pharmacologic methods have been used to reduce patients' anxiety and stabilize their vital signs before surgery and invasive procedures (11, 12). It is believed that using non-pharmacologic methods may not only decrease the patients’ BP before invasive procedures, but also reduce their need for general anesthesia (13). Several studies have shown that patient education and appropriate patient preparation would positively affect their health promotion knowledge, attitudes, skills and behaviors. It has also been shown that patient education would reduce their anxiety, increase their adaptability before surgery and could decrease the length of their hospital stay (9, 10, 14-18). However, only limited reports are available on the effects of patient preparation and its influence on vital signs for patients waiting for coronary angiography. In one of the few available studies, Buffum et al. reported that music therapy could reduce anxiety and respiratory rates in patients waiting for coronary angiography (3). In another study, the use of Benson’s relaxation technique has improved the hemodynamic status of patients undergoing coronary angiography (19). Also, Majidi has reported that the recitation of the Quran could improve the patients’ anxiety and vital signs before coronary angiography (20). All of these studies used single interventions; some of them did not report the effect of intervention on all vital signs and several others had the limitation of not being double-blinded (3).

## 2. Objectives

Considering the limited research in this area, the question remained whether a comprehensive multimodal preparation package consisting of individualized instructions, providing an educational pamphlet, showing an educational film and performing an orientation round in the angiography department could be effective in improving the patients’ vital signs before coronary angiography. Therefore, this study was designed to investigate the effects of a multimodal preparation package on vital signs of patients undergoing coronary angiography.

## 3. Patients and Methods

This matched trial was conducted on 66 patients waiting for coronary angiography. Sample size was calculated based on a study performed by Hanifi et al. (19), in which responses within each subject group were normally distributed with a standard deviation (SD) of 9.79 and the difference in the experimental and control means of 7.5. Then, it was estimated that 28 experimental and 28 control subjects are needed with a probability of 0.80 and the type I error of 0.05. However, considering an attrition rate of 20%, 33 patients were assigned in each group. The data collection instrument consisted of seven questions on demographic characteristics including age, gender, marital status, place of residence, education level, body mass index, job and a table for recording the patients’ vital signs including SBP and DBP, heart rate, respiratory rate and body temperature, that were measured using thermometers and monitoring devices. The content validity of the instruments were confirmed by several faculty members in the faculty of nursing in Kashan University of Medical Sciences and Healthcare Services, Kashan, IR Iran. The checklist reliability was tested through inter-rater reliability. To this end, two observers simultaneously completed the checklist for five patients and the results were identical. Only patients aged 30 to 70 years, referred for elective coronary angiography, who were alert and able to read Persian language and those with no previous history of coronary angiography and diagnosed psychological disorder were enrolled to the study. Exclusion criteria included active bleeding from the catheter insertion site during or after the procedure and occurrence of cardiopulmonary arrest during angiography. Sampling was done consecutively through daily referral of the second researcher to the angiography department. To prevent the contact between the intervention and control group, patients were assigned in each group on a weekly approach basis. In order to do this, patients referred in the first week of the study were randomly assigned to the control group, then, eligible patients in every other week were assigned into the control group, or the intervention group. Sampling continued until the sample size was completed.

The first measurement of vital sign was performed in a quiet room a day before angiography. Measurement of the vital signs was then repeated 30 minutes before (about one hour after implementing the preparation package in the intervention group) and 30 minutes after angiography.

### 3.1. The Preparation Package

The preparation package was offered individually to every patient in the intervention group. To do this, the second researcher was present in the angiography department during the study. Two hours before angiography, this researcher discussed the research goals with every patient. Afterwards, a 30-minute meeting was held with each patient in a quiet room, during which patients were trained about coronary angiography and the interventions before, during and after the procedure, and patients’ questions were answered. Also, an educational pamphlet about the coronary angiography was given to the patients along with some additional explanations. Then, a 10-minute video describing the angiography unit, the process of angiography, pre and post procedure care and experiences of a patient who has undergone angiography was shown to the patients. Additionally to familiarize the patient with the environment, pictures of different parts of the angiography unit and its staff were displayed for the patient. The content validity of the video and educational pamphlet were confirmed by four faculty members of the nursing school, two cardiologists and a nurse working in the angiography unit. The control group received routine training. Vital signs of this group were measured with a pattern similar to the intervention group.

### 3.2. Ethical Considerations

The Institutional Review Board and the Research Ethics Committee of Kashan University of Medical Sciences and Healthcare Services, Kashan, IR Iran, approved the study. All participants in the study signed a written informed consent form and were assured of the confidentiality of their personal information and absence of any constraint to participate in the study. Data collection was carried out after coordination with the authorities in Shahid Beheshti Hospital, Kashan, IR Iran and the coronary angiography unit.

### 3.3. Data Analysis

Data analysis was performed using SPSS V16.0 software (SPSS Inc., Chicago, IL, The USA). Kolmogorov-Smirnov test was performed to test the normality of data. Afterwards t-test was used to compare the means of vital signs parameters in the two groups. Also, Mann-Whitney U test was applied where the distribution of the data was not normal.

## 4. Results

A total number of 66 patients undergoing coronary angiography were enrolled in this study (33 in each group). A proportion of 71.2% of patients were in the age range of 51 - 70 years old. Also, 63.3% of the patients were male and all married. All patients were literate; 68.2% and 9.1% had elementary and intermediate education respectively, and 22.7% were at high school or higher levels. Additionally, 36.24% of patients were employed, 27.3% were retired and 36.4% were housewives. As the two groups were matched regarding gender, age, marital status, body mass index, education level, place of residency and occupation, no significant differences were observed.

No significant difference was observed between the mean of SBP in the intervention and control groups one day before the angiography (P = 0.82). However, a significant difference was found between the mean of SBP in the intervention and control group at 30 minutes before angiography, with values of 123.36 mmHg and 128.85 mmHg in the intervention and the control group, respectively (P = 0.03). Also, a significant difference was observed between the mean of SBP in the two groups 30 minutes after the procedure, with values of 126.45 mmHg in the intervention group and 132.85 mmHg for the control group (P = 0.03) (Table 1). 

**Table 1.** Comparison of the Mean and Standard Deviation of Systolic and Diastolic Blood Pressure, Heart Rate, Respiration Rate and Temperature in the Two Groups Measured at Three Points ^a^

Variables	Group		P Value
	Intervention	Control	
**SBP (mmHg)**			
The day before angiography	124.70 ± 13.58	125.15 ± 7.60	0.82 ^b^
30 minutes before angiography	123.36 ± 11.68	128.85 ± 6.47	0.030 ^b^
30 minutes after angiography	126.45 ± 12.44	132.85 ± 11.06	0.031 ^c^
**DBP (mmHg)**			
The day before angiography	79.11 ± 8.26	79.09 ± 6.56	0.93 ^b^
30 minutes before angiography	80.52 ± 7.20	84.58 ± 5.76	0.015 ^b^
30 minutes after angiography	79.67 ± 8.65	84.73 ± 7.30	0.023 ^c^
**Heart Rate (Bpm)**			
The day before angiography	73 ± 4.93	74 ± 6.87	0.63 ^c^
30 minutes before angiography	73.70 ± 5.33	80.24 ± 7.81	0.001 ^c^
30 minutes after angiography	72.76 ± 8.92	78.45 ± 7.36	0.01 ^b^
**Respiration Rate ** **(per minute)**			
The day before angiography	19.52 ± 1.98	19.91 ± 3.10	0.55 ^b^
30 minutes before angiography	21.82 ± 2.31	22.91 ± 2.86	0.13 ^b^
30 minutes after angiography	21.39 ± 2.71	22.18 ± 4.57	0.48 ^b^
**Temperature (°C)**			
The day before angiography	36.94 ± 0.08	36.96 ± 0.06	0.40 ^b^
30 minutes before angiography	36.95 ± 0.09	36.97 ± 0.06	0.10 ^b^
30 minutes after angiography	37 ± 0.012	37 ± 0.07	0.48 ^b^

^a^ All data are presented as Mean ± SD. Abbreviation: DBP, diastolic blood pressure; SBP, systolic blood pressure, Bpm, beats per minutes; °C, degrees centigrade.

^b^ Man-Whitney U.

^c^ T-test.

In terms of the mean DBP, no significant difference was observed between the intervention and control group one day before the procedure (P = 0.93). However, the means of DBP were significantly different in the two groups, half an hour before angiography, with values of 80.52 mmHg and 84.58 mmHg in the intervention and the control group, respectively (P = 0.015). Also, a significant difference was observed between the mean of DBP in the two groups at half an hour after the procedure, equal to 79.67 mmHg in the intervention group and 84.73 mmHg in the control group (P = 0.023) (Table 1). No significant difference was observed between the mean heart rate in the intervention and control group the day before angiography (P = 0.63). However, the mean heart rates were significantly different in the two groups, half an hour before angiography, with rates of 73.70 beat/min and 80.24 beat/min in the intervention and the control group, respectively (P = 0.001). Furthermore, a significant difference was observed between the mean heart rate in the two groups at half an hour after the procedure, with 72.76 beats/min in the intervention group and 78.45 beat/min in the control group (P = 0.010) (Table 1). In terms of the mean respiration rate, no significant difference was observed between the intervention and control group on the day before angiography (P = 0.55). Also, no significant difference was observed between the mean respiration rates in the two groups before and after the angiography (Table 1). In terms of the mean temperature, no significant difference was observed between the intervention and control group one day before the procedure (P = 0.40). Also, no significant difference was observed between the mean temperatures in the two groups before or after the angiography (Table 1). 

## 5. Discussion

In general, the findings of the current study showed that implementing the preparation package significantly improved the mean SBP and DBP in patients waiting for coronary angiography. Izadi et al. also reported that patient education through oral and written methods could significantly decrease the patients’ SBP and DBP prior to the surgical intervention (21). In another study, Hanifi et al. examined the effects of Benson relaxation techniques on anxiety in patients undergoing coronary angiography and reported that the DBP was significantly decreased in the intervention group (19). Consistent with these findings, Adib-Hajbaghery et al. (22) have also reported that massage therapy could improve SBP and DBP in patients admitted in coronary care unit. Overall, it can be concluded that appropriate preparation would improve the patients’ vital signs before coronary angiography. In the present study, implementing the preparation package could reduce the mean heart rate both before and after the procedure, in the patients waiting for coronary angiography. This finding is consistent with findings of previous studies on the effect of different patient education methods, such as video education (15), oral and written education (21), music therapy (3) and relaxation techniques (19), in patients waiting for invasive procedures, such as surgeries or angiography. Therefore, nursing interventions, such as patient education, psychological support and preparation, relaxation and music therapy, can improve the patients’ heart rate before invasive procedures like coronary angiography. These effects may be attributed to their anxiolytic impacts on patients (23).

The present study showed that after implementation of preparation package, the intervention group had lower respiration rates in comparison to the control group. However, the difference was not statistically significant. Also, no significant difference was observed between the mean body temperatures in the two groups after implementation of the preparation package. Our finding concerning respiration rate is consistent with the findings of Hanifi et al. (19), while it is inconsistent with Buffum et al. who reported that music therapy had no effects on respiration rate in patients undergoing angiography (3). Overall, it can be concluded that appropriate patient training and preparation can improve the patients’ vital signs before and after invasive procedures, as in this case, for coronary angiography. However, studies show that patients are not usually appropriately prepared for such interventions (4, 24). Therefore, most patients are still anxious and have abnormal vital signs when undergoing procedures, despite the use of different preparation methods which would increase demands for sedatives and tranquilizers before invasive procedures (3). It seems that preparation packages, such as the multimodal package used in this study, would be effective in decreasing the patients’ anxiety and stabilizing their vital signs. In conclusion, the current study showed that the preparation package was effective in decreasing SBP and DBP, as well as heart rate. Therefore, the use of multimodal comprehensive preparation packages, similar to the package used in the present study, is highly suggested. This study was conducted on a relatively small sample, and in a single center. Therefore, multicenter studies involving larger samples are suggested. Although the statistical difference was significant in BP and pulse rate, it is not clear whether it would have clinical benefits and lead to angiography complications decrease or not.
